# Aligning Ambition and Reality: A Multiple Case Study Into Synergistic Influences of Financial and Other Factors on the Outcomes of Integrated Care Projects

**DOI:** 10.5334/ijic.7736

**Published:** 2024-07-31

**Authors:** Sanne Allers, Frank Eijkenaar, Frederik T. Schut, Erik M. van Raaij

**Affiliations:** 1 Erasmus School of Health Policy and Management, Erasmus University Rotterdam, The Netherlands; 2 Rotterdam School of Management, Erasmus University Rotterdam, The Netherlands; 3 Burgemeester Oudlaan 50, 3062 PA Rotterdam, The Netherlands

**Keywords:** healthcare, integrated care, payment mechanisms, innovation, health financing

## Abstract

**Introduction:**

While the benefits of integrated care are widely acknowledged, its implementation has proven difficult. Together with other factors, financial factors are known to influence progress towards care integration, but in-depth insight in their influence on the envisioned outcomes of integrated care projects is limited.

**Methods:**

We conducted a multiple case study of four integrated care projects in the Netherlands. The projects were purposely sampled to be representative of integrated care in its different forms. A total of 29 semi-structured interviews were held with project members, both medical and non-medical staff. In addition, 141 documents were analyzed, including scientific publications and minutes of meetings. Based on elaborate project descriptions we deduced the synergistic influences of financial and other factors on the outcomes of the projects.

**Results:**

Financial factors have an important influence on integrated care projects, though this influence is neither deterministic nor isolated. This is because the likelihood of realizing a positive outcome is affected by the degree to which four key conditions are fulfilled: 1) willingness to change, 2) alignment of interests and uniformity goal, 3) availability of resources to change, and 4) effectiveness of management of external actors.

**Conclusion:**

Financial factors have an impact on the outcomes of integrated care projects and must be viewed in synergy with interrelated other factors. Crucial for realizing success in integrated care, a balance must be struck between the level of ambition set in a project and the reality of the prevailing key conditions.

## Introduction

Healthcare systems worldwide are facing increasing threats to their sustainability, including rising costs, aging populations with complex multimorbidity, and staffing shortages. These developments have resulted in a growing call for innovations in healthcare provision to alleviate the burdens on the systems [1,2]. Prominent innovations in healthcare provision involve strengthening collaboration among care providers, ensuring the right care is provided at the right place, and putting the patient at the center of care provision [3]. In other words, these innovations focus on integrated care, which is defined by Leijten and colleagues [4] as *“structured efforts to provide coordinated, pro-active, person-centered, multidisciplinary care by two or more well-communicating and collaborating care providers either within or across sectors”* (p.13).

Even though the urgent need for integrated care provision is widely acknowledged, the implementation of integrated care has proven to be difficult. The persistent fragmentations in care delivery structures result from operational complexities, regulatory challenges, obstructive cultural differences, and misaligned payment structures [2,5]. Regarding the latter, the misalignment of predominant payment structures with the general aim of integrated care can be found both in funding (e.g., temporary subsidies and grants) and in reimbursement (e.g., structural remuneration) mechanisms. Funding in the healthcare process innovation landscape has mainly focused on financing innovations with high commercial potential (e.g., innovative drugs and other medical products or treatments with a high expected financial return on investment), leaving integrated care projects without (sufficient) financial support [6,7,8]. And traditional reimbursement mechanisms, in particular fee-for-service reimbursement, tend to emphasize volume over value of care, frustrate collaboration [9,10,11,12], and contribute to fragmented care provision [13]. Nevertheless, as was concluded based on a study reviewing the different payment mechanisms for integrated care in Europe, *“there is limited evidence on the effects and effectiveness of financial incentives and other payment models in integrated care”* [14]. This conclusion was echoed in a recent systematic literature review, showing that in-depth understanding of the exact role of payment mechanisms in the integration of care is still limited [manuscript not yet in press]. Specifically, though payment mechanisms have been identified as influencing the speed and fluency of integration processes in healthcare, little is known about their contribution to the eventual outcomes of care integration projects. More generally, as concluded by Auschra [5] based on the findings of a systematic literature review on barriers for the integration of care, *“empirical research should analyze how the existence of barriers to interorganizational collaborations affects the outcome of integrated care, as barriers do not necessarily prevent or terminate collaboration, but merely slow down collaborative processes”* (p.10).

In addition, when analyzing the influence of payment mechanisms on the outcomes of integrated care, it should be acknowledged that *“when analyzing barriers (either for research purposes or in order to overcome them), it seems helpful to assume that a barrier which is visible could be caused by one or several other barriers that are not obvious at first glance”* (p.10) [5]. Therefore, a context-sensitive perspective should be adopted in which the potential interrelated influences of financial and other factors are studied. While understudied, the synergy between payment and these other factors might very well explain why financial barriers sometimes seem to be the obvious factor impeding the successful implementation of integrated care, while in other instances they seem to play only a minor role in integrating care [15].

This study aims to address this knowledge gap by investigating the facilitating and/or inhibiting influence of payment mechanisms (both funding and reimbursement, henceforth referred to as ‘financial factors’) on the outcomes of integrated care projects in the Netherlands. The influence of these financial factors will be studied alongside the influence of other, potentially interrelated, non-financial factors. We define the outcomes of these projects as the extent to which the general goal of implementing integrated care and specific objectives set in care integration projects are achieved according to those involved.

The specific objective of this study is to establish the role of financial and interrelated factors in determining the outcomes of integrated care projects. Successively, we will 1) describe the progress and outcomes of four projects attempting to integrate care processes in the Dutch healthcare system, 2) identify the factors contributing positively and negatively to the outcomes separately for each project, and 3) identify common patterns of influential factors across the projects, aiming to distill recommendations to facilitate positive outcomes of integrated care projects in practice.

## Methods

We conducted a multiple case study of innovation projects aimed at realizing integrated care across and within provider tiers. The case study methodology allowed us to construct in-depth accounts of the progress of the projects, from the perspective of the project members involved.

### Setting

The innovation projects focused on integrating care in the Netherlands. The provision of curative care in the Netherlands can roughly be divided in three tiers: a primary care tier with general practitioners acting as gatekeeper to higher tiers, a secondary care tier with general hospitals for basic care and top-clinical hospitals for more complex care, and a tertiary care tier for highly specialized care. The system for curative care is based on the principles of regulated competition with insurers and providers of care negotiating about reimbursement contracts. Ideally, this system incentivizes high quality care at the lowest possible costs [16,17]. Provider reimbursement systems are largely volume-based, though there is much variation. In hospital care, for example, closed-ended cost-per-case contracts (i.e. payments for diagnosis treatment combinations, which are similar to diagnosis related groups, under an expenditure cap) are most prevalent [18]. Healthcare providers have some amount of freedom in their choice of treatment for each patient, facilitating efficiency in care provision [19]. However, coordination of care across multiple providers is not typically incentivized.

### Case sampling

The projects were selected from the portfolio of the BeterKeten organization. For elaborate information on the strategies employed by this organization to govern the regional integration of care, see Van der Woerd et al. [20]. Over the past decade, more than 30 innovation projects have been initiated and supported by BeterKeten, each with the aim to improve quality of care through integration based on objectives formulated by medical professionals. All of these projects focus on cross-sectoral collaboration, involving multiple care organizations in the region. For this study, four projects currently in the implementation phase were purposively selected as diverse cases with the aim of being representative of integrated care in its different forms. Based on theory regarding the case study methodology, the diverse case selection technique is appropriate when sampling cases that represent different values of a predefined category [21]. This way, the cases aim to represent the full variation present. For our study, following common classifications, the projects differ in the categories of direction (i.e., horizontal and/or vertical) [22] and level of integration (i.e., linkage, coordination or full integration) [23]. Moreover, based on previous research and with assistance of the BeterKeten innovation managers, we specifically selected the cases such that they varied on the following five dimensions: 1) *the prevalence of the disease* that is focused on (i.e., high or low), 2) the *degree of change in care provision* aimed for (i.e., commonly accepted or innovative treatment), 3) the *number of care organizations* involved in the project group, 4) whether the envisioned integration is *crossing the borders of specialties*, and 5) whether the envisioned integration is *crossing tiers in healthcare provision*. Table 1 describes the selected projects in terms of these dimensions. Based on the literature, we expected that these dimensions would be associated with the size of barriers a care integration project might face and the complexity of realizing a positive outcome: a) rare diseases are less attractive to spent resources on [24], b) more extensive process change disrupts existing practice to a greater extent [25], c) a larger number of parties involved increases the chances of conflicts of interests [5], and d) crossing the borders of organizations, specialties or tiers in healthcare provision means dealing with multiple, fragmented payment mechanisms, regulations and practices [23,26]. In other words, it was expected that the variation in project dimensions would result in differences in (the size of) barriers faced in a project and the complexity of the ambition aimed for. In turn, this complexity was expected to influence the likelihood of successfully implementing integrated care in the projects.

**Table 1:** Dimensions of the different projects.


PROJECT	A	B	C	D

***Direction of integration***	Vertical	Vertical	Horizontal	Horizontal

***Level of integration***	Coordination	Full integration	Full integration	Coordination

***Prevalence of the disease***	Low	High	High	High

***Envisioned degree of change in care provision***	Moderate (commonly accepted treatment)	High (innovative treatment)	High (innovative treatment)	Moderate (commonly accepted treatment)

***Number of care organisations involved***	Six	Seven	Three	Eight

***Crossing specialties***	No	Yes	Yes	No

***Crossing tiers***	Yes (secondary and tertiary care)	Yes (primary, secondary, and tertiary care)	No (secondary care only)	Yes (secondary and tertiary care)


### Data collection

In order to identify financial, as well as other, factors contributing positively and negatively to the outcomes of the projects, we have constructed detailed accounts of the progress of each project towards realizing integrated care. To this end, we combined semi-structured interviews with document analysis. The interviews were performed with the members of the four project groups, mostly medical care providers (e.g., physicians and nurses) as well as support staff from non-medical departments of the hospitals if they had been involved in a project (e.g., IT, administrative and project management staff). For recruiting respondents via email and telephone, an overview of the project members including contact details was provided to the researchers by the project leaders. This overview was checked with each individual respondent to ensure a complete account of the people involved. Inclusion of respondents was completed upon saturation or when all available project members had been included. In total 29 interviews were held between February and June 2022, with an average duration of 48 minutes. The interviews were audio-recorded, after informed consent was provided by all respondents. The respondents were asked to reconstruct the progress within the project, including their reflections on topics such as the initiation and the objectives of the project, the steps taken to reach those objectives, the achievements, and the financial and other factors that contributed positively and negatively to the outcomes (see Appendix I for the full topic list). Finally, a member check was performed with each respondent regarding the description of their project, which resulted in several textual changes. In addition, for the purpose of triangulation, documents were obtained from the internet, the interview respondents, and the innovation managers of BeterKeten. In total 141 documents were selected, including scientific publications on several of the projects’ developments and clinical outcomes (n = 7), minutes from meetings of the project groups (n = 83), project and research proposals (n = 26), integrated medical protocols and guidelines (n = 12), questionnaires among care providers (n = 6) and applications for payment (n = 7).

### Data analysis

The interviews were transcribed verbatim by a professional transcription organization, after which all the transcripts were cross-checked with the audio file by the lead author. The interview transcripts were coded inductively using Atlas.ti 9 [27]. The coding guidelines for thematic analysis of Braun and Clarke [28] were followed, resulting in a codebook of 82 initial codes assigned to the raw data, grouped into nine generic themes such as the initial motivation, perception of achievements and barriers. Subsequently, a final analysis of the generic themes and coded data resulted in four theoretical dimensions, which will be elaborated on in the results section (see Appendix II for the codebook). Next, the document texts were coded deductively according to the nine generic themes obtained from the initial coding of the interview transcripts. The findings from the interviews were compared with the information in the documents, yielding corresponding insights.

Following this initial data analysis, elaborate project descriptions were created containing the storyline of each project, highlighting the various factors that played a role in the progress and influenced the outcomes of the projects. A summary of these descriptions per project is provided in Appendix III. Given a lack of formal project evaluations, project outcomes were assessed based on project members’ statements and documentation regarding (non-)achievement of project objectives. Specifically, a positive outcome was evaluated in terms of the extent to which the project members perceived, and project documentation reported, the following as having been realized: 1) the specific project objectives as stated by the project members and/or formulated in project documentation, and 2) the general goal of implementing integrated care (i.e., ranging from improved coordination between care providers to fully integrated care provision) [29].

It is important to note at this point that, for each project, the interview respondents also mentioned factors that had an influence on the (speed and/or fluency of the) progress of integration efforts but that – according to the respondents – did not determine the eventual outcomes of the project. Although these *process* factors were not the focus of our analysis, we do believe they provide relevant information for care integration projects, because they elucidate the context in which a project took place. Therefore, an overview of the identified process factors for each project can be found in Appendix IV.

Based on the project descriptions, we analyzed 1) whether the outcomes of the projects were positive, 2) which factors played an influential role in the outcomes of the projects, and 3) whether a relationship between financial factors and outcomes could be identified. Subsequently, a cross-case analysis was performed in which we compared the projects with more and less positive outcomes on the a priori defined project dimensions and the influential factors mentioned by the respondents. Because of our context-sensitive perspective, the analysis includes results on the synergy between the influence of financial factors with interrelated other factors. Finally, preliminary findings were discussed with the innovation managers of the BeterKeten organization to ensure validity and applicability of the results, which did not generate substantial alterations.

## Results

This section first provides a short description of each of the four projects, including the initial motivation, objectives, finances, and achievements (Table 2). Next, Section 3.2 presents an analysis of the financial and other factors that had a positive or negative influence on the project outcomes, followed by our cross-case analysis in Section 3.3.

**Table 2:** Project descriptions.


PROJECT	A	B	C	D

***Care providers in project group***	Two tertiary care and five secondary care providers treating children with a rare blood disease.	Two tertiary care providers, eight secondary care providers and two GPs treating allergies.	Two secondary care providers treating people experiencing dizziness.	One tertiary care and thirteen secondary care providers treating inflammatory bowel disease (IBD).

***Initial motivation***	High-risk patients combined with very limited knowledge in secondary care resulted in many phone calls to tertiary care.	An innovative treatment provided in secondary care could partly be given by GPs to achieve better quality for the patients and a cost reduction for society.	The disease is complex and requires multiple specialists, which resulted in patients traditionally falling through the cracks of the healthcare system.	There is a lot of regional variation in care provision, which was believed to have a negative impact on quality and/or costs of care.

***Objectives***	To improve the knowledge of providers in the region concerning this rare disease, make clear agreements regarding the referral of these patients, document a care pathway for this disease and disseminate the protocol both regionally and nationally.	To standardize the provision of the treatment amongst the different types of providers in secondary care involved in the treatment, educate GPs about the treatment, promote the transition of patients from secondary and tertiary to primary care for the continuation phase, and develop a shared EHR between the providers in all tiers.	To set up a multidisciplinary consultation hour, and to design clear triage and treatment protocols regarding the care pathway.	Overall: To increase transparency in care provision, share knowledge and expertise, to collaborate on scientific research and to improve patient information provision. Specific: To develop and implement a uniform care pathway in all hospitals in the region.

***Ambition regarding level of integration***	Low (alignment of care provision).	High (shared care provision).	High (shared care provision).	Low (alignment of care provision).

***Process duration***	One year and finalized.	Five years and finalized.	Five years and ongoing.	Four years and ongoing.

***Funding and changes in reimbursement***	No funding and no changes in reimbursement were provided.	Private funding was provided to finance the shared EHR and a project manager, but the investments were finite. Furthermore, existing reimbursement fees were inadequate to cover the costs for providers, resulting in a financial conflict of interest.	Sufficient funding was provided by the hospitals to develop and implement the project. Reimbursement agreements (a registration code and adequate fee) were made with the insurer involved. Agreements about the distribution of reimbursement within hospitals have not yet been finalized.	Private funding was provided to finance a PhD candidate, who managed the project. There were no changes in reimbursement.

***Achievements***	A regional care pathway. A guideline in the national medical manual. Regional education of providers. Information provision at a national conference. A reduced number of phone calls to tertiary care providers. Improved referral of patients. A positive experience to serve as foundation for future collaboration between these providers.	A regional care pathway. A shared EHR. Regional education of providers. Scientific publication. Not achieved: Transition of patients to primary care. Adequate reimbursement fees.	Multidisciplinary consultation hour at a joint location. A multidisciplinary meeting with additional medical specialists to discuss further treatment of multimorbid patients. Integrated registration and reimbursement structures. A website and other communication materials.	Development and measurement of shared indicators. Regional education of providers. Multidisciplinary meetings with additional specialists. Regional information provision for patients. A website and other communication materials. Scientific publications. Development of a uniform regional care pathway. Adaptation of the IT-infrastructure to the care pathway. Active dissemination of uniform care pathway. Not achieved: Implementation of uniform care pathway in daily practice of all providers.

***Outcome***	Objectives and integration reached to the level envisioned.	Neither all objectives nor integration reached to the level envisioned.	Objectives and integration were reached beyond the level envisioned.	Objectives reached to a large extent, but integration not reached to the level envisioned.


### Project descriptions and outcomes

Table 2 provides a summary of the most important characteristics per project, as well as their outcomes. More elaborate descriptions of the projects are provided in Appendix III.

### Factors influencing project outcomes

Respondents mentioned a broad range of financial and other factors that had a positive or negative impact on the outcomes of the projects. For project A, respondents stated that the rare blood disease in children and its treatment were especially suitable for designing a collaborative care pathway, because of three reasons: the manageable topic, the urgency of the problem, and the limited number of interests. As voiced by one respondent: *“Hematology is a niche area, and within it [the disease] is an even smaller area. So yeah, no one will have a problem with it. No board of directors will make a big deal out of it. So that helps”* (A2). Furthermore, many factors were mentioned that illustrate a clear direction of the project group, a great devotion and sufficient resources to make change happen, and little dependency on people outside of the project group. Although several process factors, such as limited time, complicated the progress of the project, according to the respondents these factors did not determine the project’s outcomes.

For project B, an innovative treatment provision for allergies, respondents mainly spoke about factors negatively impacting its progress and outcomes. A lack of direction, urgency, evidence, interest, as well as high levels of perceived risk and, eventually, absence of sufficient results were perceived to have harmed the participation of project members. As one respondent stated: *“In fact, I did not agree at all. And I still don’t, based on considerations regarding quality of care”* (B2). Moreover, several respondents mentioned conflicting interests due to the work and time required, and the impossibility of transferring payments in secondary care to providers in primary care. Furthermore, respondents mentioned that a lack of resources – including funding, input from medical specialists and project support – as well as the fragmentation of a large number of GPs upon whom the project depended, made it very difficult to convince the relevant actors in the region to participate in the transformation and succeed in reaching the objectives. Although several process factors that supported the project members in achieving some progress (e.g., the opportunity to obtain valuable knowledge about an innovative treatment) were also mentioned, eventually the project members were demotivated by the many setbacks and abandoned their efforts to integrate care.

For project C, the project group members, and specifically the medical specialists treating people experiencing severe dizziness, were devoted to reaching a uniform, urgent goal. The project started showing positive outcomes early in the process, strengthening the conviction that the project members were on the right track. However, the need to overcome several process barriers in this project was also mentioned, including difficulties in making agreements due to the large number of interested parties involved, a lack of time, rigid regulations, and a lack of reimbursement. Yet, the project group was able to overcome these barriers because of the high level of pre-existing trust between the parties as well as access to support staff from the hospital who were able to figure out ways to arrange innovative administration codes and financial agreements with insurers. As one respondent argued: *“I think the main reason is the presence of mutual trust, which we created together in the years before this project”* (C2). Furthermore, although the respondents mentioned a lack of interest from medical specialists outside of the project group, the project was not impeded by this barrier because it was not dependent on anyone outside the project group for realizing positive outcomes.

For project D several positive process factors were mentioned to have facilitated the project’s progress, including the access to sufficient funding and in-kind resources. Despite these facilitating factors, the project does not seem to have reached the integration it aimed for in treating people with inflammatory bowel disease. Our analysis revealed two main reasons for this. First, a limited sense of urgency, a lack of direction, and a shortfall to formulate a univocal goal among the respondents were identified. Second, the respondents mentioned high levels of professional discretion and autonomy, and therefore differences in the way specialists provide this care. As described in one document: *“The manner in which the care pathway will practically be implemented may differ per hospital. […] Because there are no strong arguments to direct this [treatment provision], the hospitals are free to decide for themselves”* (document D47). These two factors resulted in care providers being hesitant to work with a standardized care pathway, especially those who were not part of the project group.

### Key conditions for positive outcomes across projects

**Figure 1:**
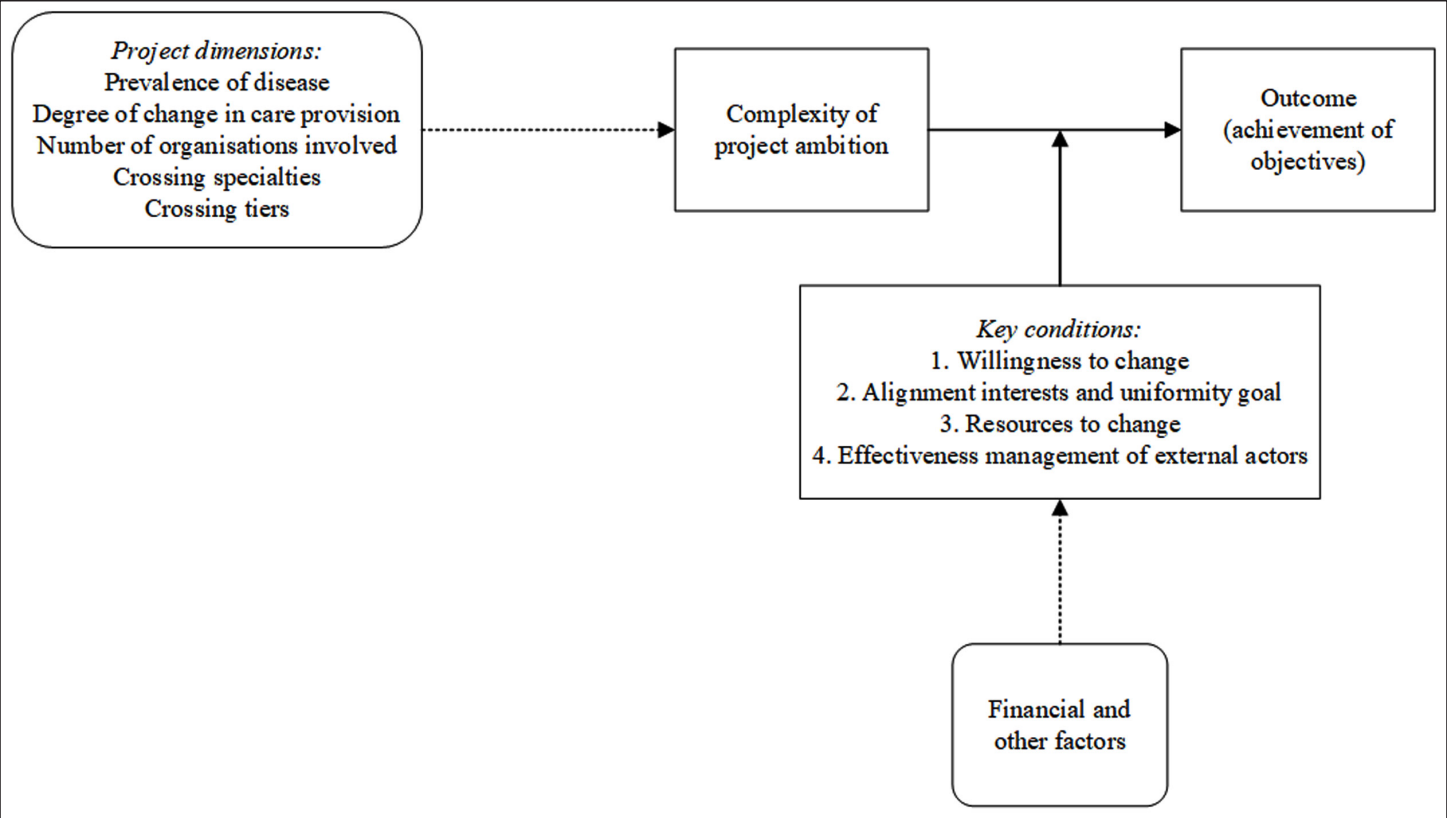
Framework on the influence of ambition and reality on the outcomes of integrated care projects.

We expected beforehand that the project outcomes would be determined by the size of the barriers faced in a project, which from previous research were assumed to be associated with the project dimensions described in Table 1. These project dimensions were expected to influence the complexity of the ambition strived for, which in turn would determine the likelihood of realizing a positive project outcome. However, our cross-case analysis did not clearly reveal the expected relationships between the project dimensions (Table 1) and the extent to which the objectives and envisioned level of integration were realized (Table 2). In other words, the project outcomes do not seem to be solely or directly determined by the prevalence of the disease that is focused on, the degree of process change aimed for, the number of organizations involved, or whether the integration is envisioned to cross the borders of specialties or tiers in care provision, nor directly by the financial factors associated with these dimensions. However, as shown by our analysis this does not mean that the project dimensions are not related to the project outcome at all. Rather, as we illustrate in Figure 1, the facilitating or hindering role of the project complexity (shaped by the project dimensions) in determining outcomes appears to be influenced.

When comparing the factors in the two projects that were evaluated more positively with the factors in the two projects that were evaluated less positively, a pattern emerges. The variety of influential factors, both financial and others (as can be seen in Table 3), are found to interrelate and can be grouped into four theoretical dimensions, henceforth called the four key conditions. This relationship of the various factors shaping the key conditions is visualized in Figure 1 with a dotted arrow. Specifically, the projects with a positive outcome are characterized by financial and other factors jointly shaping a high willingness to change, aligned interests and a univocal goal, and sufficient resources (financial, tangible, and personnel, such as dedicated medical leaders) to make the change happen. The projects with a less positive outcome were faced with a faltering willingness to change, conflicting interests diluting the objectives of different project members, and, for one project, a lack of resources. In addition, effectively managing dependency on external actors was found to be an important condition for realizing a positive outcome. In the context of this study, external actors refer to individuals who are not directly included in the project group but who are important for implementing the project. While the two projects that achieved their objectives only depended on the project members themselves, the other two projects also depended on care providers outside the project group. The data highlight the influence of these key conditions on the relationship between a more or less complex ambition and the eventual project outcome. A respondent from project C, for example, stated that *“all things, such as IT, location, financing, can eventually be arranged […] because the specialists that were involved had the will to change things”* (C2) whereas a respondent from project B stated *“the funds were gone, we had a mountain of issues to tackle, […] if we wanted to make this a success we would need a large amount of money to hire someone to lead the project, who can convince and motivate practitioners to participate”* (B1). These quotes show not only the synergistic influence of financial and other factors, but also the mitigating effect that the key conditions have on solving complex issues to realize a positive outcome.

**Table 3:** Key conditions and associated factors (financial and other) influencing project outcomes.


CONDITIONS	PROJECT A – OUTCOME POSITIVE	PROJECT B – OUTCOME NEGATIVE	PROJECT C – OUTCOME POSITIVE	PROJECT D – OUTCOME NEGATIVE

***1. Project members willing to change***	Yes Interest in the topic. Medical focus quality of care. Initial lack of knowledge about treatment. High urgency of problem due to risk of severe mistakes.	No A lack of urgency. A lack of interest. A lack of evidence. A lack of direction. Project members demotivated by many setbacks and disappointing results.	Yes Interest in the topic. Medical focus quality of care. High urgency of problem due to many patients without sufficient treatment. Results in practice early in the progress.	No A limited sense of urgency. High levels of autonomy and differentiation in treatment provision.

***2. Aligned interests and univocal goal***	Yes Singular aim. Absence of conflicting financial interests. Small niche of patients and limited number of referrals. Non-specialty-transcending treatment. Highly uniform structure of disease progression, manageable topic.	No Doubts about medical or financial aim. Conflicting interests due to the work and time involved. Insufficient knowledge and high levels of perceived risk amongst GPs. Professional GP guidelines advising against treatment. Conflicting interests due to lack of sufficient reimbursement in both tiers.	Yes Singular aim. High level of trust between parties with a univocal goal, common ground to overcome differences. Financial incentives not aligned, but no conflict of interests because parties reached an agreement.	No Failure to formulate univocal goal.

***3. Resources to change***	Yes In-kind contribution from project leads and members. Network of medical specialists. Project support BeterKeten. Time and culture to innovate in top-clinical hospitals.	No A lack of sufficient funding to maintain the EPD. A lack of sufficient funding to maintain a project manager.	Yes In-kind contribution from project leads and members. Network of medical specialists. Project manager and support from top-clinical hospital. Project support BeterKeten. Joint clinic location with essential facilities. A lot of media attention resulted in high demand innovative treatment.	Yes Sufficient funding for a PhD researcher, acting as project manager. In-kind contribution from project leads and members. IT support staff from multiple hospitals. Backing of prominent specialist academic hospital. Project support BeterKeten.

***4. Effective management of (dependency on) external actors***	Yes All medical specialists treating these patients were part of project group.	No Fragmentation of a large number of GPs, upon whom the project depended.	Yes All medical specialists and support staff needed were part of the project group.	No Fragmentation of medical specialists, upon whom the project depended.


In summary, the project dimensions determine the complexity of the project ambition, which does impact the likelihood of realizing a positive outcome. However, as shown in our analysis, the impact of a given level of complexity on the likelihood of a realizing a positive outcome is influenced by the degree to which each of the four key conditions is fulfilled. The more facilitating financial and other factors are present in a project, the higher the degree of fulfillment of the key conditions. In that case, an exceedingly complex ambition can be turned into a positive outcome. Ultimately, to increase the likelihood of realizing a positive outcome in integrated care, the level of complexity of a project ambition needs to be aligned with the reality of the prevailing key conditions.

## Discussion

We conducted a multiple case study of four integrated care projects to identify the influence of financial and other interrelated factors on the outcome of realizing integrated care in practice.

### Key findings

Our analysis revealed three key findings. Firstly, although we could not confirm the expected direct relationship between financial factors and the likelihood of realizing a positive outcome (as suggested by previous literature), these factors do have an important influence on integrated care outcomes. There are financial factors influencing the degree to which key conditions for positive outcomes are fulfilled, affecting the likelihood of realizing a positive outcome. Although financial factors have an important influence on the outcomes of integrated care projects, this influence is neither deterministic (i.e., the influence can be mitigated or strengthened by the degree to which key conditions are fulfilled) nor isolated (i.e., the influence interrelates with the impact of other factors). This explains why, in practice, addressing financial barriers will not always result in the successful implementation of integrated care, and why persistent financial barriers will not always obstruct its implementation. Nevertheless, given that diminishing financial barriers will increase the likelihood of realizing integrated care, it is important to maintain ongoing efforts in devising better payment mechanisms such as bundled payment models for full disease pathways or integrated capitation payments [30].

Secondly, our multiple case study revealed four key conditions that influence the likelihood of realizing a positive outcome: willingness to change of medical and non-medical staff, alignment of interests and uniformity of goal, availability of resources to change and effectiveness management of external actors. Previous literature has identified the separate influences of these elements on the implementation of integrated care [31,32,33,34,35]. Importantly, however, as our analysis shows, none of these conditions are deterministic. They need to be viewed in relation to one another, as well as in their alignment with the complexity of the ambitions aimed for. González-Ortiz et al. [36] concluded from their literature review on research about the dimensions of integrating care that the results of that research *are mostly lists of key building blocks to integrated care, rather than frameworks supporting the process of implementation*. We believe our findings do provide such a framework that can support the practice of integrating care, acknowledging the synergistic influences of financial and other factors. In section 4.2, we specify how to make these findings actionable in practice.

Thirdly, we find that project outcomes are eventually determined by the alignment of the degree of fulfilment of the key conditions to integrate care and the complexity of the project ambition. When the willingness to change is high, interests are aligned with a shared univocal goal, resources are available and all actors are on board, ambitions can be high and complex. To understand the implementation of integrated care, it is essential for future research to adopt a balanced perspective in which influencing factors are studied simultaneously to understand their joint impact on outcomes [5]. Moreover, for integrated care to succeed, a similarly comprehensive perspective needs to be adopted in practice in which practitioners acknowledge the essential balance between ambition and reality.

### Aligning ambition and reality

**Figure 2:**
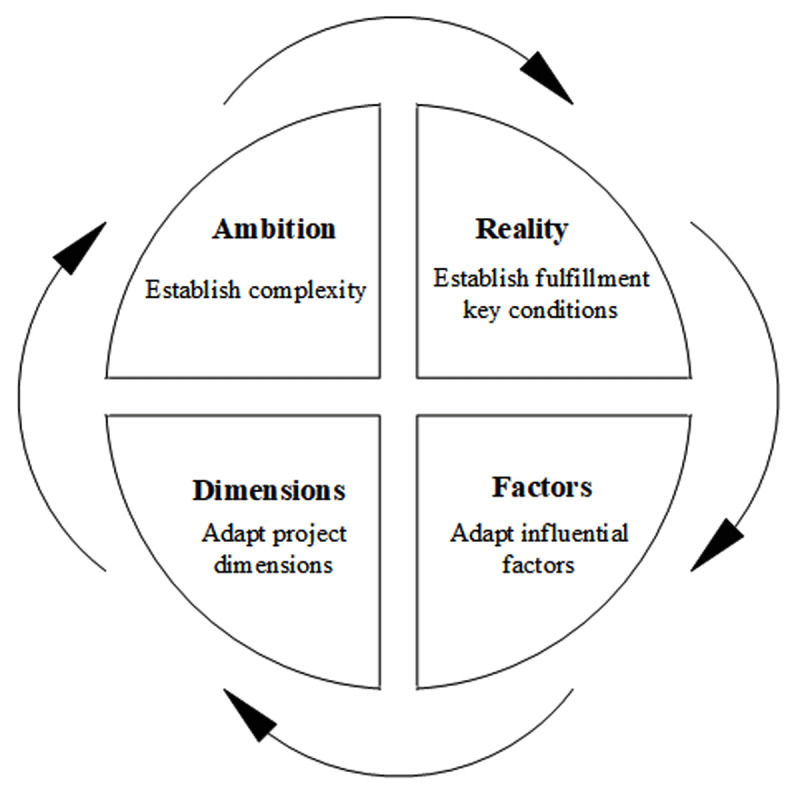
Aligning ambition and reality cycle.

We propose a four-step iterative cycle to help succeed in aligning ambition and reality in practice (Figure 2). To start, the complexity of the ambition can be established by assessing the project dimensions, such as the ones explicated in Table 1 or other relevant dimensions. Second, the degree of fulfillment of the key conditions can be established by evaluating factors that may act as barriers or facilitators. Third, in case there is misalignment between the ambition and reality, project members can work on further fulfilling the conditions by resolving inhibiting factors and/or strengthening facilitating factors. Finally, in case the misalignment persists, the complexity of the ambition can be reduced by adapting the dimensions of the project. It is important to acknowledge the iterative and continuous nature of this alignment process. If conditions change or factors are adapted during the course of a project, the project dimensions and ambition should be adapted accordingly.

### Limitations

Although we believe that our findings apply broadly to integrated care projects in healthcare, we must consider the potential impact of the context in which these specific projects took place. All projects were embedded in the context of the BeterKeten organization. In addition to the project support provided at no additional costs, three characteristics of this context favor a positive outcome. First, in this context, medical professionals typically come up with the initial plan and take the lead in the development and implementation of the project. Previous research has shown the indispensability of medical professionals with a high willingness to change when introducing process innovations [37]. Our research builds on this conclusion by highlighting other conditions that need to be fulfilled to put these medical professionals in a position to make change happen. Second, the studied projects are all projects in which top-clinical secondary and/or tertiary hospitals played a role: care organizations with a mandate for research and development of innovation. This context has been shown to be a vital part of successful collaboration projects because the leaders in these organizations offer room for innovation and in-kind contributions from staff members [38]. Third, each project aimed for integration of organizations in the same region. The physical proximity of the integrating parties is likely to affect the likelihood of positive outcomes, as this is positively related to a sense of cohesion and trust between the parties [39].

In addition to the potential impact of the specific BeterKeten context, there are several other potential limitations which are related to our study design. First, a bias could result from the variation in the time between the end of the project and our moment of data collection. Some projects had very recently finished the project or were in the process of finishing up and were still very positive about the changes it would bring. Other projects had finished up a few years ago and could have been more negative because the change they initially envisioned was not realized in practice. Third, the reliance on self-evaluation of the respondents regarding the achievement of project objectives could have introduced bias in assessing the outcomes of the projects. However, we identified no contradictions in outcome evaluation between the interview data and (official) project documentation, diminishing the risk of recollection or self-evaluation bias. Finally, all projects encountered chance events that, according to the respondents, had an impact on the project outcomes. For example, the project manager in project B became ill and was not able to finish her work, and the establishment of a joint clinic in project C was advanced by the hospitals because their previous shared hospital location was closed. While such chance events will have had some impact on the progress in the projects, respondents stated it was the more generalizable factors, as depicted in Figure 1, which eventually determined the project outcomes.

## Concluding remarks

Financial factors have an important influence on the outcomes of integrated care projects, but this influence is neither deterministic (i.e., the influence can be mitigated or strengthened) nor isolated (i.e., the influence interrelates with other factors). In addition, a balance must be struck between the level of ambition set in a project and the reality of the prevailing key conditions. This balance should henceforth be adopted as a prime focus of both researchers and care integrators in creating a better understanding and realization of integrated care in practice.

## Additional Files

The additional files for this article can be found as follows:

10.5334/ijic.7736.s1 Appendix I. Semi-structured topic list.

10.5334/ijic.7736.s2 Appendix II. Codebook.

10.5334/ijic.7736.s3 Appendix III. Project descriptions and factors influencing the outcomes.

10.5334/ijic.7736.s4 Appendix IV. Influential process factors in each project.
