# Supplementary material for: Aligning Ambition and Reality: A Multiple Case Study Into Synergistic Influences of Financial and Other Factors on the Outcomes of Integrated Care Projects
Source: Int J Integr Care. 2024 Jul 31;24(1):11. doi: 10.5334/ijic.7736 (PMC11295916; doi:10.5334/ijic.7736)
Supplement: Appendix I. — Semi-structured topic list. [file ijic-24-3-7736-s1.pdf]

Appendix I. Semi-structured topic list

This topic list serves to facilitate semi-structured interviews. Depending on the specific role and knowledge of the respondent, certain aspects can be given more or less attention.

1) <u>Introduction</u>
   a. Personal introductions: interviewer and respondent
   b. Short introduction to the innovation project (*all subsequent questions concern the experiences of the respondent with this particular project*)

2) <u>Process in general</u>
   a. Would you be able to give a general description of the innovation process? Including the start and the current state of the innovation? (Initial motivation, duration, pace)

3) <u>Objectives</u>
   a. What are the objectives aimed for in this project?
   b. What is the ultimate goal of this project in your opinion? In other words, when do you think the project is a success?

4) <u>Achievements</u>
   a. Which achievements have been realized in the project?

5) <u>Status of implementation integrated care</u>
   a. Which professionals were informed about the integrated care pathway?
   b. Which professionals provide care according to the integrated care pathway? Do you provide care according to the integrated care pathway?
   c. Why do you/don't you provide care according to the integrated care pathway?
   d. Does the integrated care pathway align with your current/previous way of working? Does the pathway apply to the patients you treat?
   e. Which concerns do you have regarding the provision of care following the integrated care pathway?

6) Facilitating factors
    a. Which factors have had an important influence on the achievements realized in the project?

7) Barriers
    a. Which factors have been the largest barriers in developing and implementing the project?

8) Support
    a. What are things that helped you overcome these barriers, to the extent you managed to overcome them?

9) Finances
    a. Which forms of financing did this project receive?
        i. How would you describe the process of securing this financing?
    b. Which forms of financing did this project apply for, but not receive?
        i. What were the most important reasons for this, in your opinion?
    c. Which forms of financing would you like to receive for this project in the future?
        i. Why? What would happen if you do not succeed in securing these types of payment?
    d. How would you describe the influence of finances on the project?

10) Stakeholders
    a. Which stakeholders are/were involved in the project?
        i. What is/was the role of these stakeholders?
    b. Are there any stakeholders you miss/ have missed in the project?
        i. What would be the additional value of these stakeholders?

11) Cooperation
    a. How was the cooperation between the different stakeholders?
    b. What was the impact of the project on the interaction between professionals within this organisation?

i. What was the impact of the project on the interaction between doctors and nurses?

c. What was the impact of the project on the interaction between professionals from different organisations?

12) Concluding remarks

a. Any further questions or remarks related to the interview?
