# Supplementary material for: Aligning Ambition and Reality: A Multiple Case Study Into Synergistic Influences of Financial and Other Factors on the Outcomes of Integrated Care Projects
Source: Int J Integr Care. 2024 Jul 31;24(1):11. doi: 10.5334/ijic.7736 (PMC11295916; doi:10.5334/ijic.7736)
Supplement: Appendix II. — Codebook. [file ijic-24-3-7736-s2.pdf]

Appendix II. Codebook

Applied code	**Generic theme**
• Motivation project	Initial motivation
• Characteristic diease	Characteristics
• Objective	Objectives
• Duration project<br>• First meeting<br>• Involvement medical professional<br>• Involvement manager<br>• Involvement patient association<br>• Perception of progress	Progress
• Reach project<br>• Implementation in practice<br>• Results<br>• Evidence measurement	Achievements
• Perceived value	Value
• Perception of results<br>• Cooperation between tiers<br>• Cooperation project group<br>• Job description medical professional<br>• Future	Perception of achievements

- Barrier: agreements, autonomy, covid, fragmentation cost-benefit, fragmentation suppliers, fragmentation medical professional, lack of evidence, lack of reimbursement, lack of funding, lack of interest, lack of knowledge, lack of stakeholders, lack of support, lack of results, lack of direction, lack of time, lack of devotion, lack of urgency, lack of trust, IT infrastructure, small peripheral hospital, competing interests, strategical interests, regulations, medical guidelines, risk, tradition.
- Finances
- Missing stakeholders
- Staff turnover
- Interests salaried versus independent employment

→ Barriers

- Facilitator: (free) project support, adaptive abilities, academic/top-clinical hospital, well-known person, BeterKeten, covid, compliancy, facilities, physical meetings, joint location, lack of knowledge, absence of financial interests, clarity finances, in-kind contribution, interest, IT infrastructure, medical leadership, quality, media attention, medical content, personal network, manageable topic, results, aligned strategy, devotion, tradition, trust, univocal goal, urgency.
- Finances
- Finances did not change
- Finances topic of conversation

→ Facilitators
