# Supplementary material for: Aligning Ambition and Reality: A Multiple Case Study Into Synergistic Influences of Financial and Other Factors on the Outcomes of Integrated Care Projects
Source: Int J Integr Care. 2024 Jul 31;24(1):11. doi: 10.5334/ijic.7736 (PMC11295916; doi:10.5334/ijic.7736)
Supplement: Appendix III. — Project descriptions and factors influencing the outcomes. [file ijic-24-3-7736-s3.pdf]

Appendix III. Project descriptions and factors influencing the outcomes.

1. Project description A

Project A is a collaboration project between care providers from secondary and tertiary care, focusing on treatment for a rare blood disease (immune thrombocytopenia) in children. The objectives of the project were to improve the knowledge of providers in the region concerning this rare disease, make clear agreements regarding the referral of these patients, document a care pathway for this disease and disseminate the protocol both regionally and nationally. The project group consisted of two providers from tertiary care and five providers from secondary care who met six times over the course of a year. Objectives reached in this project include a regional care pathway, also recorded as a guideline in the national medical manual, regional education of providers, information provision at a national conference, a reduced number of phone calls to tertiary care providers, and a positive experience to serve as foundation for future collaboration between these providers. The respondents were very positive about the outcomes, and stated *"we did not have to overcome major obstacles"* (A6). All in all, this project showed how integrating care can be as easy as child's play: the objectives were reached, and the goal of integrating care was achieved to the level that was initially envisioned.

1.1 Description of financial factors project A

The project received no funding for development or implementation, apart from the resources of the BeterKeten organization, yet this was not perceived as harming the progress. The work done for the innovation project was perceived to be a part of the job description of medical professionals, even though it did require effort and time outside office hours. In terms of reimbursement, not a lot has changed in practice. Due to the small number of patients that were moved between providers, the low level of integration, and the involved providers being paid a fixed salary, conflicts of financial interests were absent. In contrast, the respondents felt the project produced feelings of clarity and security about finances. Secondary care providers gained the opportunity to treat patients for a longer period of time in addition to knowing exactly at what point tertiary care providers should refer patients back to them. Tertiary care providers were happy to spend their time only treating the most complex patients, given that they had their hands full with them anyways.

1.2 Analysis of influential factors within project A

In retrospect, the respondents stated that the disease and its treatment were especially suitable for designing a collaborative care pathway, because of three reasons. First, the highly uniform structure of the disease progression with only a few treatment options was easy to document. Second, the rarity of the disease combined with several very risky complications doubled the risk of severe mistakes and consequences. *"There is little experience in the peripheral hospitals: therefore, diagnosing these patients is difficult. The pediatricians in the academic hospital are only at the end of the line, and then it turns out there is a more serious issue"* (document A4). *"In case of a rare disease, for which experience is not quickly gained and at times fatal bleeding can occur, it is good to collaboratively document the treatment in a care pathway"* (document A5). Third, the non-specialism-transcending treatment and very small niche of patients resulted in a limited number of involved parties and interests. *"Hematology is a niche area, and within it [ITP] is an even smaller area. So yeah, no one will have a problem with it. No board of directors will make a big deal out of it. So that helps"* (A2).

In addition to the manageable topic, the urgency of the problem, and the limited number of interests, we identified four factors that contributed to the achievements of this project. First, there was a willingness to change among the members of the project group. This willingness to change came from an interest in the topic, a medical focus on the quality of care provision, an initial lack of knowledge on the topic, and a general devotion to the project group as well as the urgency previously mentioned. Second, the willingness to change was directed towards the same goals. The respondents made note of a singular aim and an absence of any conflicting financial interests. The project was even perceived to clarify and better protect interests. Moreover, the number of patients was so small that the actual shift is relatively small, hence does not trigger large financial interests. *"Of course, it had to do with the fact that there are no shifts of large numbers of patients, there were no financial interests. And that's what makes it interesting for the medical specialists, the focus was very much on the medical content"* (A2). Third, the project had the resources to facilitate the change. These included the project support from BeterKeten, in-kind contribution from the project lead and project members who perceived this task as part of their job description, the network from the medical specialists, and the freedom to innovate in the involved top-clinical hospitals. Fourth, even though the respondents mentioned a lack of interest outside of the project group, the project was not impeded by this barrier because it did not need anyone outside the project group to participate in order to realize positive outcomes. On the other hand, several factors

were mentioned to have complicated the progress of the project: a lack of time to schedule meetings, a lack of support in PR, and one project member being from a small peripheral hospital where innovation was facilitated less. Even though these factors might have impacted the process, they did not influence the eventual outcomes.

2. Project description B

Project B is a collaboration project between care providers in primary, secondary and tertiary care, focusing on an innovative treatment for allergies (immunotherapy). The treatment consists of two parts; the first, short-term initiation phase and the second, continuation phase which lasts for 3 to 5 years. At the start of the project, the treatment was used by different types of healthcare providers in secondary and tertiary care. However, it remained relatively unknown amongst general practitioners (GP) in primary care. Moreover, the continuation phase was in primary care perceived as being labor intensive, complex, and as having rare but very severe risks. In addition, the effectiveness was questioned by several respondents. The aims of this project were to standardize the provision of the treatment amongst the different specialties in secondary care involved in the treatment, educate GPs in primary care about the treatment, promote the transition of patients from secondary and tertiary to primary care for the continuation phase, and develop a shared electronic health record (EHR) between care providers in all tiers. The project group consisted of eight providers from secondary care, two professors from tertiary care and two GPs, who met four to five times per year over the course of five years. The respondents mainly spoke about setbacks the project endured during these years: the project progressed for too long, the collaboration between care specialists from different tiers was arduous, and it proved difficult to recruit GPs to partake in the project group. Eventually, the respondents were disappointed with the lack of uptake of the treatment among the GPs. Moreover, several respondents mentioned the scope of the project kept broadening which made it even more difficult to successfully reach the objectives set. Consequently, not all of the project's objectives were reached, and the goal of integrating care was not achieved to the level that was envisioned.

2.1 Description of financial factors project B

The respondents mentioned significant financial barriers in the project, both inadequate funding and insufficient reimbursement were perceived to have played a role. The project received funding from private companies, with which to finance the development of the shared EHR and the appointment of a project manager. However, the funding was finite. After the funding was depleted, the maintenance costs of the EHR technology could no longer be financed and the work of the project manager was too intensive to be taken up by the providers themselves. The local hospitals, government and healthcare insurers were contacted, yet none were prepared to invest in the integration project. Furthermore, the

existing reimbursement fees for the treatment were not sufficient to cover the costs incurred for providing the treatment in secondary or tertiary care. Hence, moving part of the treatment to primary care could result in savings. *"So if you could organize it in a way that, well, those patients could go from the hospital to the GP. For the costs we make [and the reimbursement we receive], care can easily be arranged in primary healthcare. However, the transition of those finances is not easy to arrange"* (B1). In primary care, no reimbursement fee could be made available for providing the treatment and it was not possible to transfer money from the reimbursement available in secondary care to providers in primary care. *"Moving immunotherapy from secondary to primary care entails challenges in the area of reimbursement. The current fee for an extended consultation provides little incentive for the GP to take on this intensive task"* (document B1). These financial disincentives to take up the provision of the treatment resulted in suspicions from several project members regarding the underlying reasons for the project. On the one hand, respondents reported that the motivation to align and integrate care provision between medical professionals inspired the project. On the other hand, respondents mentioned that the actual motivation reason was likely to be financial.

2.2 Analysis of influential factors within project B

Four factors were identified to have hindered the project in reaching the objectives that were set. First, the willingness to change was not shared by all project members. Respondents mentioned a lack of direction, urgency, evidence, interest, and, eventually, results to have harmed the participation of project members. In addition, it was experienced to be difficult to arrange meetings due to a lack of time. Insufficient knowledge and high levels of perceived risk, combined with professional guidelines advising against the treatment, were said to reduce the willingness to change under GPs. *"Lack of specific knowledge regarding allergies in primary care and concerns regarding safety, turned out to be important obstacles in the past to shift patients for the continuation phase to the GP"* (document B4). *"In fact, I did not agree at all. And I still don't, based on considerations regarding quality"* (B2).

Second, the involved parties mentioned conflicting interests. The work, time and risks involved with the continuation phase resulted in some reluctance on the side of primary, secondary, and tertiary care providers to take up the treatment. *"How can I create support in my hospital [for this treatment]? It takes time and effort, for which we will not be reimbursed. Naturally, stakeholders are not very eager"* (document B13). Moreover, the innovative treatment would require the GPs to put in much effort to be educated about the

procedure. An even more important source of conflicting interests was the financial disincentive, due to a lack of sufficient reimbursement for both tiers. The project members had approached both the national government as well as individual healthcare insurers, to ask for financing of the integration. However, as one respondent argued, *"this condition does not have priority for payers"* (B1), so no reimbursement was made available. The issue of contradicting financial incentives resulted in suspicions from several project members regarding the underlying reasons for the project and allergic reactions to the proposed integration plans.

Third, the project was impeded by a lack of resources, including funding, medical specialists, and project support, to realize change. The project received some funding from a pharmaceutical company to pay an IT company to create the shared EHR and finance the salary of a project manager. But, after all, the funding was finite, and after some time the project lost its resources. Maintenance costs for the app, as well as the rights to implement it, were taken over by the IT company. The work performed by the project manager was too much to be done by the medical specialists themselves. Missing this driving force significantly hurt the project. Respondents emphasized the negative influence of fragmentation between costs and benefits of healthcare innovation. According to one respondent, this kind of innovation is rarely financed upfront by the government or insurers, because the savings from the innovation should pay for the investments needed to realize change. However, as he explained the catch-22, "*without the savings there is no sufficient funding to realize those savings in the first place*" (B1).

Fourth, the fragmentation of a large number of GPs upon whom the project depended, made it very difficult to convince everyone in the region to participate in the transformation and succeed in reaching the objectives.

On the other hand, there were several factors that supported the project members in achieving some progress. First, the respondents positively recounted the project support from BeterKeten. Second, the general lack of knowledge on this innovative treatment created the opportunity to add something valuable. Third, the efforts and interest from the project lead and members of the project team were greatly valued by the respondents. Yet, eventually, the project support from BeterKeten ended, the project members were demotivated by the many setbacks, and they abandoned their efforts to integrate care.

3. Project description C

Project C is a collaboration project between care providers from two secondary care hospitals, focusing on an innovative diagnostic method for people experiencing dizziness. Despite the commonality of this disease, patients often fall through the cracks of the traditional healthcare system. The disease is complex, with high levels of multimorbidity, and setting a diagnosis requires a lengthy examination based on listening to the patient's life story. Traditionally, this required the patient visiting multiple hospital departments and a lot of deliberation between specialists. The innovative approach proposed in this project consists of a lengthy, multidisciplinary consultation including different specialists in which the patient can be diagnosed timely and referred to the proper treatment. A holistic approach towards the patient is key to this innovation. Accordingly, the aims of this project were to set up a multidisciplinary consultation hour, and to design clear triage and treatment protocols regarding the care pathway. The motivation for the project can be found in the personal interest of one of the project leads, who saw a similar innovative approach towards the disease in another region of the Netherlands. Hence, the start of this project was described by one of the respondents as *"a hobby that got out of hand"* (C5). The project had been ongoing for five years, and the project members had expanded from solely two secondary care providers to a group with support staff from project management, communication, administrative, and financial departments, and management of the hospitals. The respondents were very positive about the achievements, including a multidisciplinary consultation hour at a joint location, a multidisciplinary meeting with additional medical specialists to discuss further treatment of multimorbid patients, an integrated administration and reimbursement structure, a website and other communication materials. All in all, the respondents indicated that the project turned out to be a dazzling success: the objectives were reached above and beyond the initial vision of the project leads, and the goal of integrating care was achieved.

3.1 Description of financial factors project C

After the initial start of the multidisciplinary consultation by the care providers, the project was picked up by the hospitals' management. They decided to award the project the status of value-based healthcare project, in line with the hospitals' strategy, which made available support staff from the hospitals and investments from the innovation payments received from insurers. This way, minimal financial barriers were experienced for the development and implementation of the project. Contrarily, the most difficult part of the integration project

was experienced to be agreeing on reimbursement arrangements for the present and the future sustainment of the project. Initially, costs were divided fifty-fifty between the two providers and the consults were registered as a regular consult with one medical specialist. However, the amount of reimbursement received for a regular consult did not cover the costs of the longer consultation with multiple specialists. In the absence of appropriate financial and administrative agreements between the two hospitals and with the insurers, a financial conflict of interest threatened the sustainability of the project. *"It is stated that the financial prospect must be established before the collaboration can commence"* (document C16). However, when the project gained access to support staff from the hospitals, there was a possibility to negotiate an agreement with the healthcare insurers about a DRG-registration code and an appropriate reimbursement fee. Therefore, the financial conflict of interest was resolved and the project is currently sustainably implemented. For the future, however, the hospitals are bound by national agreement to zero growth in production. Agreeing on extra reimbursement for innovative treatments automatically results in fewer room to provide other, regular, treatments. *"The response given by insurers is always: 'It's fine if you want to perform more of this, but you have to look internally what you can do less'. It is kind of a waterbed effect"* (C2). The current limits on the number of patients that can be seen in the dizziness center have resulted in long waiting lists and a difficult choice for the hospitals about which treatments they will continue to provide. Moreover, the innovative treatment takes up a lot more time, resulting in fewer total patients treated hence lower income for the departments involved. Therefore, even though financial agreements have been made between the hospitals and insurers, agreements about the distribution of reimbursement within the hospitals had not been finalized yet. Nevertheless, all respondents were convinced this issue will be solved.

3.2 Analysis of influential factors within project C

Four factors have been identified to have added to the achievements of this project. First, the project group members, specifically the medical specialists, were devoted to change. They expressed an interest in the topic, praising the focus on improving quality of care provision and acknowledging the urgency of the problem. Moreover, the project started showing results in practice very early in the process, strengthening the conviction that the project members were on the right track. Second, the people involved in the project were aiming for a univocal goal and the respondents mentioned that the envisioned innovative treatment was reachable. Financial interests were eventually not experienced to hinder the project substantially,

because the specialists themselves stated to have no interest to get involved in financial discussions and the financial departments of the hospital managed to reach agreements with most of the parties involved. Finally, respondents mentioned that there was a high level of pre-existing trust in the hospitals and different departments wanting to reach the same goal, so the financial agreements that had not been reached yet were expected to follow soon. *"I think the main reason is the presence of mutual trust, which we created together in the years before this project. It is multifaceted: trust among the medical specialists, who really know each other very well which makes you believe that also on the content you will figure out a way, and trust between the financial departments. […] You really notice if that trust is absent or very brittle, the smallest issue arises and you are immediately set a few steps back or the project comes to an end"* (C2). Despite potentially severe financial barriers along the way, the project managed to prevent a conflict of interest. Third, this project had abundant resources to make change happen. The resources started with the in-kind contributions of the project leaders and their network, followed by the project manager and (financial) support from a top-clinical hospital and eventually a joint clinic location with essential facilities. In addition, the media attention resulted in a high demand for this treatment, making it even more of a success. Fourth, even though the respondents mentioned a lack of interest from medical specialists outside of the project group, the project was not impeded by this barrier because it did not rely on anyone outside the project group to participate in order to realize positive outcomes.

On the other hand, there were some barriers to overcome in this project, including difficulties in making agreements due to the large number of interested parties involved, a lack of time, a lack of knowledge, rigid regulations, and a lack of reimbursement. Yet, the project group was able to overcome these barriers because of the high level of trust and devotion between the parties, and the access to support staff from the hospital who were able to figure out ways to arrange innovative administration codes and financial agreements with insurers.

4. Project description D

Project D is a collaboration project between care providers from secondary and tertiary care, focusing on pharmaceutical treatment with biologicals for inflammatory bowel disease (IBD). The aim of this project is to develop and implement a uniform care pathway for this treatment across the region. Apart from this general understanding, respondents' views diverged concerning the specific objectives of the project. Several respondents were convinced that there was potential to improve the quality of care by reducing regional variation in outcomes and costs. Or the quality of care could be improved by reducing within-hospital variation caused by different generations of specialists in hospitals who adhere to different treatment pathways. Other respondents simply stated that reaching uniformity in treatment was the goal in itself. Another motivation mentioned was to develop a minimal care pathway that prescribes a basic level of care all patients are entitled to. Contrarily, a motivation could have been to reduce the activities in the treatment to prevent unnecessary care. A respondent mentioned the motivation for the project was to reduce costs and improve cost-effectiveness, because *"it's what we always have to focus on"* (D11). There were also respondents who stated the care pathway was developed for research reasons, in order to be better able to compare the effects of future innovations in the treatment. Finally, one of the project members argued there was no specific reason behind the project but *"the project just existed"* (D7). The project group consisted of a PhD candidate, a medical specialist from tertiary care, thirteen providers (both medical specialists and nurses) from secondary care and IT support staff from the involved hospitals. The project had been active for four years, starting from the commencement of the PhD candidate, and achievements mentioned by the respondents included developing a uniform regional care pathway that potentially could help to reduce care activities; adaptation of the IT-infrastructure; and several scientific publications. Nonetheless, most respondents expressed doubts about the benefits of the project, the scope of collaboration and standardization in practice, and the actual changes made in the care provided. Many respondents felt a standardized care pathway was hard to swallow thus they deviated from the pathway at their own discretion. Other respondents even admitted they did not (consciously) follow the pathway at all. Moreover, the care pathway had been adapted to the wishes of every hospital, resulting in an absence of real standardization. As the objective was described in one of the ambition documents: *"Standardization of IBD care within the region with due observance of differentiation of the hospitals"* (document D22). *"The care pathway was adjusted to the local context as to not disrupt local processes"* (document D2).

In conclusion, there are strong question marks whether this project has integrated care sufficiently. Thus, although the objective to develop and disseminate a uniform care pathway was realized in this project, implementation in practice was only partially successful and most respondents state that the goal was not achieved to the level that was envisioned.

4.1 Description of financial factors project D

The respondents described the effort they had to make to get sufficient funding for a PhD candidate, who could manage the project and study the results. The project team eventually managed to organize sufficient funding and the research project could start. Financial support was not required for other aspects of the project, the IT staff was on the payroll of the participating hospitals and the care providers stated that innovation of care was part of their responsibility as care provider. Furthermore, the project had no significant impact on reimbursement amounts because adjustments to the care provision were limited. In addition, most of the respondents perceived the financial aspect of the project as the responsibility of the financial departments.

4.2 Analysis of influential factors within project D

Three types of facilitating factors were identified. First, the project members had an interest in the topic from a medical perspective and they were positive about the opportunity to gain knowledge on a medical topic with limited evidence. Also, the proposed care pathway was perceived as manageable and providers also praised its adaptability to each hospital. Second, respondents mentioned there were no conflicts of interest, because financial interests did not have an influence (no major changes in care provision hence no major changes in reimbursement amounts) and the respondents trusted the other project group members who they had been collaborating with for many years. Third, the project had access to resources to make change happen. These resources included the funding for a PhD researcher, who also acted as project manager, project support from BeterKeten, the project leaders and group members in-kind contribution, the possibility to use the available expertise of different departments of specialists of an academic center, and the IT support staff of the participating hospitals.

Despite these facilitating factors, the project does not seem to have reached their goal of persuading every care provider to work with a uniform pathway. The main factors identified as impeding this progress are twofold. First, a limited sense of urgency, a lack of direction, and a shortfall to formulate a univocal goal among the respondents were identified.

Second, the respondents mentioned high levels of professional discretion and autonomy, and therefore differentiation in the manner in which individual medical specialists provide this care. As described in one document: *"The manner in which the care pathway will practically be implemented may differ per hospital. […] Because there are no strong arguments to direct this [specific ways to provide the treatment], the hospitals are free to decide for themselves"* (document D47). These two factors resulted in care providers being hesitant to work with a uniform care pathway, especially those who were not part of the project group. In addition, respondents also mentioned that other obstacles were a lack of time, smaller hospitals that are less able to support innovation, and rigid privacy regulations regarding data sharing.
