# Supplementary material for: Aligning Ambition and Reality: A Multiple Case Study Into Synergistic Influences of Financial and Other Factors on the Outcomes of Integrated Care Projects
Source: Int J Integr Care. 2024 Jul 31;24(1):11. doi: 10.5334/ijic.7736 (PMC11295916; doi:10.5334/ijic.7736)
Supplement: Appendix IV. — Influential process factors in each project. [file ijic-24-3-7736-s4.pdf]

Appendix IV. Influential process factors in each project.

Table I. Influential factors (financial and other) influencing the progress of each project but not determining the eventual outcomes.

**Project A**	**Project B**	**Project C**	**Project D**
Barriers that were overcome<br>• A lack of time to schedule meetings.<br>• A lack of support in PR.<br>• Small peripheral hospitals facilitating innovation less.	Facilitators that were not sufficient<br>• General lack of knowledge about this innovative treatment created the opportunity to add something valuable.<br>• In-kind contribution from the project lead and members of the project team.<br>• Project support BeterKeten.	Barriers that were overcome<br>• Difficulties making agreements due to many parties involved.<br>• A lack of time to schedule meetings.<br>• A lack of knowledge about the possibilities to embed innovations structurally.<br>• Rigid regulations.	Facilitators that were not sufficient<br>• General lack of evidence and guidelines created the opportunity to add something valuable.<br>• Proposed integrated treatment perceived to be reachable.<br>• Treatment adaptable to local circumstances.<br>• Absence of financial or other interests, due to limited changes in care provision.<br>• High level of trust between project members.<br>• Sufficient resources for change.

Classification: Internal
